# The Different Effects of Firsthand Pain and Nonpain Electrical Stimulation on Pain Empathy: An EEG Study

**DOI:** 10.1155/prm/9676653

**Published:** 2025-10-10

**Authors:** Ping Chen, Baoying Chen, Wei Feng, Bofeng Zhao, Zhiqiang Pan, Junya Mu, Ming Zhang

**Affiliations:** ^1^Department of Medical Imaging, The First Affiliated Hospital of Xi'an Jiaotong University, West Yanta Road, Xi'an 710061, China; ^2^Imaging Diagnosis and Treatment Center, Xi'an International Medical Center Hospital, Xitai Road, Xi'an 710100, China

**Keywords:** electroencephalograph, firsthand pain, pain empathy, pain sensitivity

## Abstract

**Background:**

Empathy for pain is a complex psychological process that enables us to understand the feelings of others experiencing pain. Personal pain experiences may shape our empathetic responses, though the precise relationship between empathy for pain and pain perception is not fully understood.

**Objective:**

This study investigates how firsthand pain experiences influence brain responses related to empathy for pain.

**Methods:**

We recruited 26 participants who underwent either painful or nonpainful electrical stimulation. They were then shown static pictures of hands in painful and nonpainful situations and asked to report their subjective ratings of pain empathy. Electroencephalograph activity was recorded to analyze the neural basis of their experiences.

**Results:**

Participants who experienced painful stimulation reported higher empathy levels when viewing painful images. Electroencephalograph data revealed that painful stimulation elicited larger N1 and P2 amplitudes at the Cz electrode compared to nonpainful stimulation. It also enhanced N2 amplitudes in the frontal-central region when viewing painful pictures. Nonpainful stimulation reduced the discriminatory ability of N2 for subsequent painful and nonpainful images. Correlation analysis showed that individuals with higher pain sensitivity had greater variation in P2 responses to painful and nonpainful stimuli but less variation in N2 responses related to pain empathy.

**Conclusions:**

These findings suggest that experiencing pain directly may enhance brain responses to others' pain, thereby increasing levels of pain empathy. This enhancement may be influenced by individual pain sensitivity and is less pronounced in those with high pain sensitivity.

## 1. Introduction

Pain empathy is a complex phenomenon that allows the observer to understand and share other-pain sensory and emotional qualities [1, 2]. Observers perceive the pain of others through their individual representations, which are formed from their own pain experience, so that empathy grows with familiarity with the situation [3]. According to the “shared representations” theory, empathy for others' pain is based on the same psychological and neural representations that underlie firsthand pain (subjectively experienced pain) [4, 5]. In addition, both preclinical and clinical studies have consistently demonstrated that patients with persistent or chronic pain generally show more explicit pain empathy when they witness suffering in others [6, 7]. These findings suggested that pain empathy, a component of pain that can be influenced by psychological factors, is often closely linked to individuals' pain experiences. However, the neural mechanism of how the pain experience affects pain empathy remains largely unclear.

Recently, the combination of the priming paradigm and electroencephalogram (EEG) technology has emerged as an intuitive and representative method for investigating the specific relationship between pain empathy and pain [8, 9]. For example, subjects are initially presented with a picture depicting people experiencing either pain or nonpain, and then they are exposed to a notional stimulus, an auditory stimulus, or a tactile stimulus themselves. By analyzing the changes in subjects' event-related potential (ERP) and perceptual rating, the selective activation of pain empathy on firsthand pain representations is highlighted. These studies allow for initial observations of a specific correlation between pain empathy and firsthand pain scores [10]. Conversely, researchers have also examined how sensory priming affects pain empathy. They found that self-pain priming predicted a slightly faster response and a smaller ERP amplitude to other people's pain targets compared to nonpain priming, while responses to nonpain targets were unaffected [8]. However, to the best of our knowledge, few studies have assessed the neurological interaction between individual variations in firsthand pain and the subsequent empathetic response to another person's pain. Employing the priming paradigm to collect brain oscillations of self-pain and other-pain consecutively within a single trial could provide further understanding of the impact of firsthand pain on empathy towards pain.

In this study, we hypothesized that individual differences in the brain's response to firsthand pain would influence subsequent brain responses to pain empathy. To test our hypotheses, we conducted a within-subject two-factor (stimulation ∗ picture) experiment. During each trial, participants received either painful or nonpainful electrical stimulation of their little finger, followed by a series of visual stimuli comprising painful or neutral pictures of the hand. At the same time, 32-channel EEGs and participants' empathy ratings for the pictures were recorded.

## 2. Methods

The research aims and experimental procedures had been fully explained to all participants, and the written informed consent of all participants was obtained. All studies were conducted in accordance with the Declaration of Helsinki and were approved by the Institutional Review Board of the First Affiliated Hospital of the Medical College of Xi'an Jiaotong University (No. 2017-154).

### 2.1. Participants

Twenty-eight healthy participants were recruited from the local community. Exclusion criteria were: (1) individuals with any physical illness, such as a brain tumor, hepatitis, or epilepsy as assessed according to clinical evaluations and medical records; (2) those with the existence of chronic pain conditions (e.g., tension-type headache, fibromyalgia, etc.); (3) those with the existence of a neurological disease or psychiatric disorder; (4) pregnancy; (5) those using prescription medications within the last month; (6) those with alcohol, nicotine, or drug abuse.

### 2.2. Questionnaires

All participants completed a series of standardized questionnaires to assess their baseline psychological traits related to pain. These measures were administered in validated Chinese versions, with scoring procedures based on the original manuals. To evaluate fear of pain, participants completed the Fear of Pain Questionnaire (FPQ), which captures responses across three types of situations: minor, severe, and medically related [11]. Higher scores reflect greater fear across these domains. The Pain Catastrophizing Scale (PCS) was used to measure negative thought patterns in response to pain, including persistent worry (rumination), exaggeration of threat (magnification), and feelings of helplessness [12]. Elevated scores indicate stronger tendencies toward catastrophic thinking. Pain-related anxiety was assessed using the Pain Anxiety Symptoms Scale (PASS), which examines cognitive, physical, and behavioral signs of anxiety triggered by pain [13]. Higher scores suggest greater anxiety sensitivity in pain contexts. Finally, the Pain Sensitivity Questionnaire (PSQ) estimated each participant's perceived sensitivity to everyday painful experiences [14]. Individuals with higher scores reported greater subjective sensitivity to pain. These trait measures were subsequently analyzed in relation to behavioral responses and EEG data to explore individual differences in pain processing.

### 2.3. Stimulus and Experimental Design

**Figure 1:**
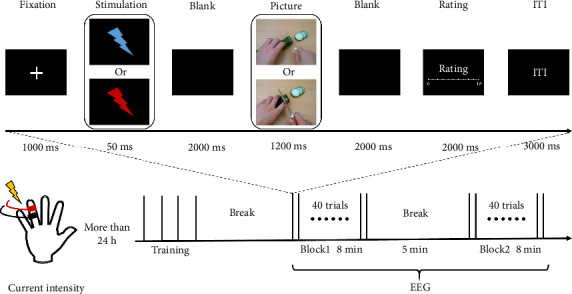
Experimental procedures.

Figure 1 gives an overview of our experimental design, including current intensity measurement and EEG acquisition.

#### 2.3.1. Current Intensity Measurement

At least 24 h before the EEG recording, pain sensitivity was measured by generating constant-current square-wave electrical pulses through an electrical stimulator to give nociceptive stimulus (pulse duration: 50 ms; SXC‐4A, Sanxia Technique Inc., China). The electrical pulse travels through a pair of surface electrodes placed on the upper side of the proximal phalanx of the left ring finger. The intensity of the stimulation was calibrated specifically for each participant: the initial intensity was 300 μA, and the electrical stimulation was gradually increased with the step length of 100 μA (up to 9900 μA), until the participants' numerical rating scale (NRS; 0 = no pain, 10 = unbearable pain) score for the intensity of the same stimulus reached 2 or 6 three times in a row. The current intensity corresponding to NRS 2 was defined as a low-intensity stimulation, representing a clearly detectable sensation without pain (e.g., tingling or pressure). The current intensity corresponding to 6 will be used as a painful stimulation. This intensity represents a suprathreshold pain level for participants, ensuring the elicitation of a clearly perceptible pain sensation.

#### 2.3.2. EEG Acquisition

Participants were instructed to sit in a comfortable chair in a silent, temperature-controlled room (28°C) and try to relax. To solicit pain and pain empathy from participants, a stimulation ∗ picture paradigm with electrical stimulation and static visual stimulation was used. And the similar paradigm had been previously used in recent studies [8, 10]. The participants' pain and nonpain sensations were elicited by applying electrical currents of intensities corresponding to 6 and 2 on the surface electrode located on the upper side of the proximal phalanx of the left ring finger. Furthermore, we employed static visual stimulation selected from the International Affective Picture System to evoke participants' empathic responses to pain. Specifically, we utilized a stimulation set comprising 40 pictures, all depicting hand conditions in everyday scenarios and categorized into two groups: painful and nonpainful pictures. The painful pictures (e.g., hand cuts resulting from improper knife usage, totaling 20) and nonpainful pictures (e.g., proper knife usage for chopping vegetables, also 20 in number) were carefully matched in visual attributes such as brightness, contrast, and color to ensure consistency across stimulation conditions. All pictures were uniformly sized at 7.08 inches wide by 5.32 inches tall, with a pixel density of 100.

In training and testing phase, pictures were presented at the center of the computer screen with a black background, and participants maintained a viewing distance of approximately 80 cm from each picture. The specific experimental procedure is illustrated in Figure 1: At the beginning of each trial, a fixation point was displayed for 1000 ms; subsequently, the fixation point disappeared, and an electrical stimulation of either 6 or 2 intensity was immediately applied for 50 ms. During this period, only a pure black background was visible on the screen, which persisted for an additional 2000 ms after the electrical stimulation ended. Following this, either a painful or nonpainful picture was presented for 1200 ms. After the picture presentation, there was a 2000-ms blank screen interval. Subsequently, participants were required to verbally rate the unpleasantness they felt while viewing the picture on an 11-point numerical rating scale within the next 2000 ms (with 0 indicating no discomfort and 10 indicating unbearable discomfort). It should be emphasized that the unpleasantness rating should reflect the participant's direct emotional response to observing the picture, rather than a speculation about the painfulness evoked by the depicted situation. Each trial was separated by an interval of 3000 ms.

Each EEG recording block comprised 40 trials, interspersed with a 5-min in-between blocks rest period. To balance the order effects across different blocks, we employed the following strategies: (1) Both blocks utilized the same set of 40 pictures, each comprising 20 pairs of images depicting painful and nonpainful scenarios. These images were randomly associated with either painful or nonpainful electrical stimulation resulting in four distinct picture types. (2) Within the two blocks, the type of electrical stimulation paired with a particular image varied. (3) The order of the blocks was reversed for consecutive participants.

To maintain the randomness of trials and prevent the repeated occurrence of similar stimuli, we employed a pseudo-random arrangement of all images within each block. This ensured that no single image type would appear more than three times consecutively. In the training phase, participants were asked to complete a task comprising four trials. Each trial presented a different image type, allowing participants to familiarize themselves with the experimental procedure. It is important to note that the images used in these training trials were distinct from those used in the testing phase.

During the entire EEG acquisition process, participants were guided to sustain their attention, fixate their gaze on the central point of the computer screen, and minimized head and body movements. This was to prevent excessive eye movements and electromyographic signals that could interfere with the EEG reading. Furthermore, participants were required to stay awake and concentrate on perceiving the stimuli presented throughout the experiment.

### 2.4. EEG Recording and Preprocessing

EEG data were recorded by TMSi (the Netherlands) SAGA 32-channel EEG recording equipment and its supporting software Polybench. We adhered to the internationally recognized 10–20 system and precisely placed 32 electrodes on the participants' scalps, using the tip of the nose as an online reference point. The frequency range of the band-pass filter was set to 1–100 Hz, and the sampling rate was configured at 1000 Hz. Prior to data collection, thorough checks were conducted to guarantee that the impedance of all EEG electrodes remained below 10 kΩ.

EEG data were preprocessed using EEGLAB [15], an open-source toolbox running in the MATLAB environment (R2021a; MathWorks, USA). The Reference Electrode Standardization Technique (REST) [16] was used for re-reference and band-pass filtered between 1 and 30 Hz (Basic FIR filter, cutoff frequency (−6 dB): [0.5 30.5] Hz). Then, EEG epochs were extracted using a window analysis time of 1500 ms (500 ms before and 1000 ms after the onset of the stimulation). Epochs contaminated by gross artifacts (e.g., large muscle activity) were removed. Meanwhile, the participants' ratings of the pictures at this epoch were also deleted. Across all participants, the number of epochs excluded during preprocessing was 5.2 ± 4.7 (mean ± SD), with a range of 1 to 10 epochs. Meanwhile, the participants' ratings of the pictures at this epoch will also be deleted. Independent component analysis (ICA) was performed using EEGLAB's runica algorithm to identify and remove components associated with ocular artifacts (blinks, saccades) and muscle noise. Components were classified based on spectral power topography, temporal profiles, and dipole locations, with non-neural components (e.g., high-frequency muscle activity or frontal eye fields) discarded. Participants' ratings corresponding to rejected epochs were excluded from further analysis [17]. Based on re-examination and statistics across all participants, the number of independent components removed during ICA preprocessing was 4.6 ± 1.2 (mean ± SD), with a range of 3–7 components.

### 2.5. EEG Feature Extraction

#### 2.5.1. ERPs

We performed trial averaging under the same conditions to generate six stable time-locked average waveforms for each participant. Based on the topographical distribution of potentials within 0–1000 ms post-stimulus, Cz was selected as the key electrode for analysis in the painful and nonpainful stimulation conditions. For four types of pictures, we chose the average value from Fz, FC1, FC2, and Cz as the data for the electrode of interest. Similar to previous studies, we extracted the average amplitudes of the N1 and P2 components induced by electrical stimulation at the Cz electrode [18], as well as the average amplitudes of the N1 and N2 components induced by picture stimulation at the Fz, FC1, FC2, and Cz electrodes [19, 20], for subsequent statistical analysis and comparison.

#### 2.5.2. Spectral Power

We combined the data from two types of pictures after the same electrical stimulation and divided the baselines at the electrodes of interest into two categories: baselines after painful stimulation and baselines after nonpainful stimulation. Discrete Fourier transform was then applied to these baselines, and the power spectral density (PSD) was calculated. The power spectrum of different frequency bands represents the power distribution of the signal in a specific frequency band. We used PSD to calculate the spectral power over the four frequency bands for each participant (delta/*δ* 1–3 Hz, theta/*θ* 4–7 Hz, alpha/*α* 8–13 Hz, and beta/β 14–30 Hz).

### 2.6. Statistical Analyses

To investigate the differences between painful and nonpainful stimulation, we conducted a paired-samples *t*-test on their ERP components, including N1 and P2, as well as the subsequent baseline power. Additionally, to ensure that the data conformed to the assumption of a normal distribution, we performed a logarithmic transformation (log10) on the EEG spectral power and current intensity.

To explore the effects of electrical stimulation and picture stimulation on empathy for pain responses, we conducted a two-way repeated-measures ANOVA on the empathy ratings corresponding to the four types of pictures, as well as the N1 and N2 components of ERP.

To comprehensively understand the relationships between pain-related scale scores, empathy for pain ratings, EEG features induced by electrical stimulation, and EEG features induced by picture stimulation, we employed Pearson correlation analysis and mediation analysis methods. All mediation analyses were performed in SPSS software, and the confidence intervals were verified using 5000 bootstrap samples to ensure the robustness of the results.

## 3. Results

### 3.1. Demographic Characteristics

**Figure 2:**
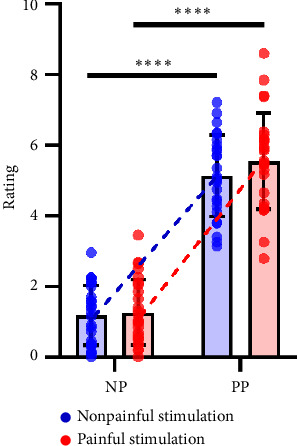
Two-factor repeated-measures ANOVA results. Empathy ratings across the four picture types (PP, NP, PN, NN). Significant main effects of electrical stimulation and picture type, as well as their interaction, were observed. ^∗∗∗∗^*p* < 0.0001.

A total of 28 healthy participants were recruited. However, two participants were excluded due to incomplete experimental procedures and/or serious data contamination. This resulted in a final sample of 26 healthy participants (age: 22.0 ± 0.6, 5 females, 21 males).  Table 1 provides a comprehensive overview of their demographic and behavioral characteristics, as well as their scores on pain-related questionnaires. A paired *t*-test showed a significant difference (*t*(25) = 18.33, *p* < 0.001) in the mean values of log-transformed current intensities between painful stimulation and nonpainful stimulation. In addition, we conducted a two-factor repeated-measures ANOVA to assess differences in empathy ratings under the four types of pictures. The result is shown in Figure 2 with the significant main effect of electrical stimulation (*F*(1, 25) = 7.66, *p*=0.011) and picture stimulation (*F*(1, 25) = 242.70, *p* < 0.001), as well as their interaction effect (*F*(1, 25) = 4.95, *p*=0.035). To further study these effects, we conducted a simple effect analysis and used paired *t*-tests to assess the differences under various conditions. As can be seen from Table 2, painful stimulation amplifies an individual's rating of painful pictures, but it did not influence their rating of nonpainful pictures.

### 3.2. Comparison of ERP Under Different Electrical Stimulations

**Figure 3:**
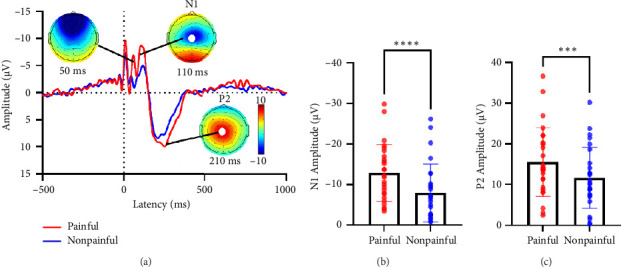
ERP under electrical stimulation. ERP at the Cz electrode encompassing N1 and P2 components along with electrical stimulation noise (a). Paired-sample *t*-tests for N1 and P2 (b). ^∗∗∗^*p* < 0.001, ^∗∗∗∗^*p* < 0.0001.

As shown in Figure 3(a), the ERP response to electrical stimulations at the Cz electrode during the earlier latency period exhibits two opposite components: N1 and P2. These components are primarily concentrated in the central region and are significantly influenced by electrical stimulation within a 50-ms window. Paired *t*-tests analysis revealed that, in comparison to nonpainful stimulation, the absolute amplitudes of both N1 (*t*(25) = −5.97, *p* < 0.001, Figure 3(b)) and P2 components (*t*(25) = 4.59, *p* < 0.001, Figure 3(c)) showed a significant increase under painful stimulation.

### 3.3. Comparison and ANOVA in EEG Under Different Picture Stimulation

For the comparison between mean baselines after painful (0.2 ± 0.3 μV) and nonpainful (0.3 ± 0.2 μV) stimulations, no statistical result was found. In addition, paired *t*-tests were conducted to compare the average power across the four frequency bands in the picture baselines after the two types of electrical stimulations. Significant differences were only observed in the delta band, where the average power was markedly higher after painful stimulation (*t*(25) = −2.15, *p*=0.042, Supporting Figure S1).

**Figure 4:**
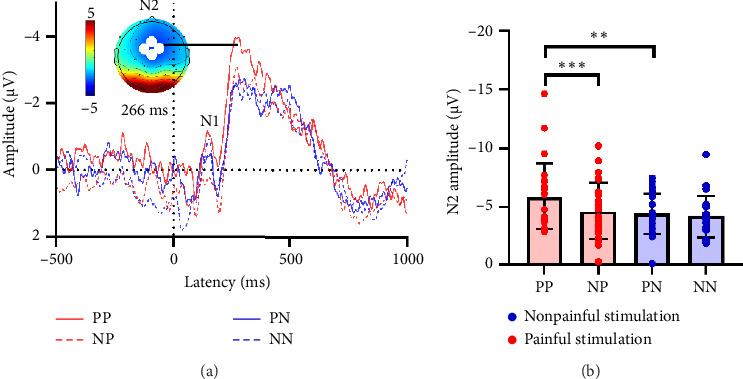
Event-related potentials (ERPs) elicited by picture stimulation. (a) Grand-averaged ERPs at Fz, FC1, FC2, and Cz, showing N1 and N2 components under the four picture conditions. (b) Simple effects analysis of N2 amplitudes. Pairwise comparisons are shown (PP vs. PN, NP vs. NN, PP vs. NP, PN vs. NN), highlighting the specific contrasts that account for the significant interaction effect. ^∗∗^*p* < 0.01, ^∗∗∗^*p* < 0.001.

As shown in Figure 4(a), during the earlier latency period, the ERP responses to picture stimulations at Fz, FC1, FC2, and Cz electrodes primarily exhibit two distinct negative components N1 and N2. These components are predominantly observed in the frontal and central regions. It is noteworthy that the amplitudes of the N1 component do not show significant differences across the four types of pictures. However, a different pattern emerges when we examine the N2 component using a two-factor repeated-measures ANOVA. There is a significant main effect of electrical stimulation (*F*(1, 25) = 8.91, *p*=0.006), a significant main effect of picture stimulation (*F*(1, 25) = 9.75, *p*=0.004), and a significant interaction effect between electrical and picture stimulations (*F*(1, 25) = 4.76, *p*=0.039). To further study these effects, we performed a simple effect analysis (Figure 4(b) and Table 3). A paired *t*-test revealed that painful stimuli could amplify individuals' N2 responses to painful pictures, but had no effect on nonpainful pictures.

### 3.4. Correlation Analysis of Electrical Stimulation Phase and Picture Stimulation Phase

**Figure 5:**
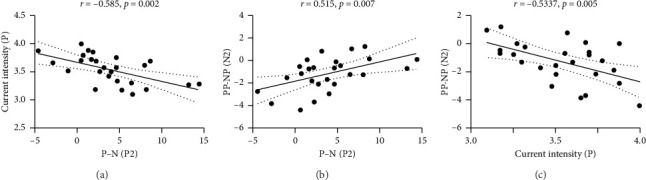
Correlation analysis. The variation in P2 amplitudes for different electrical stimulations is negatively correlated with the intensity of painful electrical current (a). The variation in N2 amplitudes for painful pictures after different electrical stimulations is positively correlated with the variation in P2 (b). The variation in N2 is negatively correlated with the intensity of painful electrical current (c); P = painful electrical stimulation, N = nonpainful electrical stimulation, PP represents painful picture after painful stimulation, NP represents painful picture after nonpainful stimulation.

Correlation analysis revealed a negative correlation between the variation in P2 amplitude, which were elicited by painful and nonpainful stimulation and the intensity of the painful electrical current experienced by individuals (*r* = −0.59, *p*=0.002, Figure 5(a)). Additionally, the variation in N2 amplitude, which were elicited by painful pictures after different electrical stimulations, positively correlated with the variation in P2 elicited by electrical stimulation (*r* = 0.52, *p*=0.007, Figure 5(b)). The variation in N2 amplitude also negatively correlated with the intensity of the painful electrical current (*r* = −0.53, *p*=0.005, Figure 5(c)). However, when we conducted a subsequent mediation analysis to explore the relationships among the intensity of the painful electrical current, the variation in P2 amplitude, and N2 amplitude, we did not find a statistically significant relationship.

## 4. Discussion

By combining painful electrical stimulation with empathy-inducing picture stimulation, our study employed EEG analysis to investigate the impact of firsthand pain on empathy for pain in the brain. We obtained three main findings. First, painful stimulation increased individual's ratings of pictures depicting a person experiencing pain. Second, during the electrical stimulation, painful stimulation elicited higher absolute amplitudes of N1 and P2 components at Cz compared to nonpainful stimulation. Third, painful stimulation increased individual's N2 components at Fz, FC1, FC2, and Cz for pictures depicting a person experiencing pain. This suggests that individuals with higher pain sensitivity showed smaller changes in N2 amplitude when viewing painful pictures after receiving painful stimulation. In summary, our findings suggested that firsthand painful electrical stimulation amplified empathy for pain, potentially through an increase in N2 amplitude. The degree of this increase may be linked to individual pain sensitivity.

### 4.1. Individual Pain Experience Is a Significant Factor Influencing Empathy Ratings for Pain

Our behavioral results confirmed that painful electrical stimulation significantly increased empathy ratings for painful pictures, but not for nonpainful ones. This suggested that individuals may become more sensitive to pain after experiencing painful stimuli. Recent studies supported this, suggesting that changes in pain perception could affect one's understanding of painful situations, thereby modifying their empathy towards others' pain [21, 22]. These changes manifest in both subjective ratings and physiological responses. For example, in patients with microvascular angina, double-blind cardiac pain stimulation led to significantly higher pain incidence and lower pain thresholds during true stimulation compared to sham stimulation, intensifying pain perception [23]. Our findings suggested that painful stimuli may heighten individuals' attention and interpretation of pain-related information. When confronted with painful pictures, they may concentrate more on the painful elements, temporarily amplifying their empathy for pain. However, this selective attention and interpretation are not triggered by nonpainful pictures.

### 4.2. Painful Stimulation Can Lead to Differences in Empathy-Related EEG Responses

The ERP induced by nociceptive stimuli includes several components, notably N1, N2, and P2. It is proposed that N1 may represent the brain's discriminative processing of external stimuli, while P2 might reflect the brain's subsequent processing, interpreting, and generating corresponding responses to pain information [24, 25]. EEG studies indicated that painful stimuli typically amplify the amplitude of N1 and P2. These changes may be related to individuals' perception, attention, and emotional responses to pain [26, 27]. In our study (Figure 3(a)), we observed that both painful and nonpainful stimulation resulted in peak N1 and P2 amplitudes at the Cz electrode location. Our results are consistent with previous studies, which observed that harmful electrical stimulation leads to an increase in the amplitude of N1 and P2.

Research has revealed that the brain's baseline activity can influence the efficacy of subsequent stimulation [28]. In our findings, the baseline delta power following painful stimulation was higher than that after nonpainful stimulation. Our previous study observed the close association between delta power in the prefrontal cortex and individual differences in empathy for pain [29]. Hence, the findings of the current study suggested that painful electrical stimulation might alter participants' baseline activity, thereby impacting their empathetic capacity towards pain.

In line with empathy ratings, our analysis of ERP evoked by picture stimulation showed a significantly greater absolute amplitude of the N2 component for painful pictures after painful stimulation compared to those after nonpainful stimulation. This finding supported our hypothesis that the brain's response to firsthand painful stimulation may influence empathy for pain. Numerous studies have supported this perspective, suggesting that personal pain experience may activate brain regions associated with empathy and pain processing [4]. The activation of these regions could potentially enhance an individual's empathetic expression during pain empathy responses [30]. Notably, our results showed that differences among various pictures persist after painful stimulation, but disappear after nonpainful stimulation. Since this change was unrelated to baseline, we speculated that nonpainful electrical stimulation may affect the brain's sensitivity to subsequent picture stimulation. Taken together, our findings suggested that the effects of different types of electrical and picture stimulation on brain activity during empathy for pain might be complex and dynamic. Painful electrical stimulation can enhance observers' empathetic responses to painful pictures, while nonpainful electrical stimulation may affect the brain's sensitivity to subsequent stimulation.

### 4.3. Pain Sensitivity Affects the Degree of Empathy Enhancement by Painful Electrical Stimulation

Our research found a negative correlation between the P2 variation in ERP between painful and nonpainful stimulations and the intensity of painful electrical current. This suggested that individuals with higher pain sensitivity might exhibit a more pronounced increase in the P2 component when transitioning from nonpainful to painful stimulation. Typically, P2 and N2 components are associated with attentional and emotional processing [31]. It suggested that individuals with high pain sensitivity may be more susceptible to pain-related stimuli, possibly allocating more attentional resources and experiencing stronger emotional reactions [32, 33].

Additionally, we observed that the N2 variation among painful pictures after different electrical stimulations was also negatively correlated with current intensity. But given that N2 is a negative component, this finding suggested that individuals with higher pain sensitivity are less influenced by painful stimuli when developing pain empathy. However, despite a positive correlation between the variations of P2 and N2, we have not identified their mediating effects, nor obtained results related to the differences in baseline power influenced by painful and nonpainful stimulations. Therefore, we inferred that high pain sensitivity in individuals may not directly enhance empathic responses by increasing their pain reactions. Instead, it may reduce the impact of firsthand painful stimulation on empathy for pain. This could be due to withdrawal or avoidance behaviors stemming from survival mechanisms, where a heightened concern for others' pain in individuals with high pain sensitivity may lead to diminished reactions to others' pain as a result of retreating or avoiding their own pain [34]. Nevertheless, this does not alter the facilitating effect of firsthand pain on empathy for pain. In essence, our findings suggested the coexistence of the shared representation theory [35] of empathy for pain and the threat value of pain hypothesis [34].

## 5. Limitation

There are several issues that should be discussed in future research. Firstly, the sample size is limited, which may not adequately represent the general population. The low number of participants could potentially lead to insignificant findings in correlation analysis or introduce incidental outcomes. Furthermore, our participants are primarily recruited from a university campus, with issues such as gender imbalance and limited diversity in age and cultural backgrounds, potentially limiting the applicability and generalization of the research findings. Secondly, there are concerns regarding causality inference and ecological validity. While this study identifies an association between electrical pain stimulation and empathy response, establishing causality may require more rigorous experimental designs, such as manipulating pain stimulus intensity to observe variations in outcomes. Additionally, research conducted in laboratory settings may not fully reflect real-life situations, as pain experiences in everyday contexts can be more complex and varied.

## Figures and Tables

**Table 1:** Characteristics of all participants.

Characteristics	Participants
Age (years)	22.0 ± 0.6
Sex (female/male)	5/21
Rating of PP	5.5 ± 0.3
Rating of NP	5.1 ± 0.2
Rating of PN	1.3 ± 0.2
Rating of NN	1.2 ± 0.2
Current intensity (P)	3.5 ± 0.0
Current intensity (N)	3.0 ± 0.1
FPQ	90.6 ± 2.7
PCS	21.4 ± 1.5
PASS	43.7 ± 2.3
PSQ	73.3 ± 3.0

*Note:* Data were reported as mean ± standard error unless otherwise indicated. PP represents painful picture after painful stimulation, NP represents painful picture after nonpainful stimulation, PN represents nonpainful picture after painful stimulation, and NN represents nonpainful picture after nonpainful stimulation; FPQ refers to the Fear of Pain Questionnaire, PCS refers to the Pain Catastrophizing Scale, PASS refers to the Pain Anxiety Symptoms Scale, and PSQ refers to the Pain Sensitivity Questionnaire.

**Table 2:** The simple effects analysis on rating.

Pair	*t*	df	Significance	Cohen's *d*
PP vs. PN	14.95	25	< 0.001	1.46
NP vs. NN	15.12	25	< 0.001	1.34
PP vs. NP	3.03	25	0.006	0.69
PN vs. NN	0.91	25	0.370	0.46

*Note:* PP represents painful picture after painful stimulation, NP represents painful picture after nonpainful stimulation, PN represents nonpainful picture after painful stimulation, and NN represents nonpainful picture after nonpainful stimulation.

**Table 3:** The simple effects analysis on N2.

Pair	*t*	df	Significance	Cohen's *d*
PP vs. PN	−3.48	25	0.002	2.20
NP vs. NN	−1.41	25	0.170	1.80
PP vs. NP	−4.26	25	< 0.001	1.48
PN vs. NN	−0.61	25	0.548	1.92

*Note:* PP represents painful picture after painful stimulation, NP represents painful picture after nonpainful stimulation, PN represents nonpainful picture after painful stimulation, and NN represents nonpainful picture after nonpainful stimulation.

## Data Availability

The data and code that support the findings of this study are available from the corresponding author upon reasonable request.

## Supporting Information

Supporting Figure S1 displays comparative baseline power spectral analysis of electrical stimulation modalities across neural frequency bands (*δ*, *θ*, *α*, *β*), with statistical significance indicators.
