# Supplementary material for: The Different Effects of Firsthand Pain and Nonpain Electrical Stimulation on Pain Empathy: An EEG Study
Source: Pain Res Manag. 2025 Oct 10;2025:9676653. doi: 10.1155/prm/9676653 (PMC12534147; doi:10.1155/prm/9676653)
Supplement: Supporting Information — Additional supporting information can be found online in the Supporting Information section. [file 9676653.f1.docx]

**Supplementary Material**

An additional figure showing the baseline power difference between the two types of electrical stimulation is available as a supplementary file uploaded with this submission.


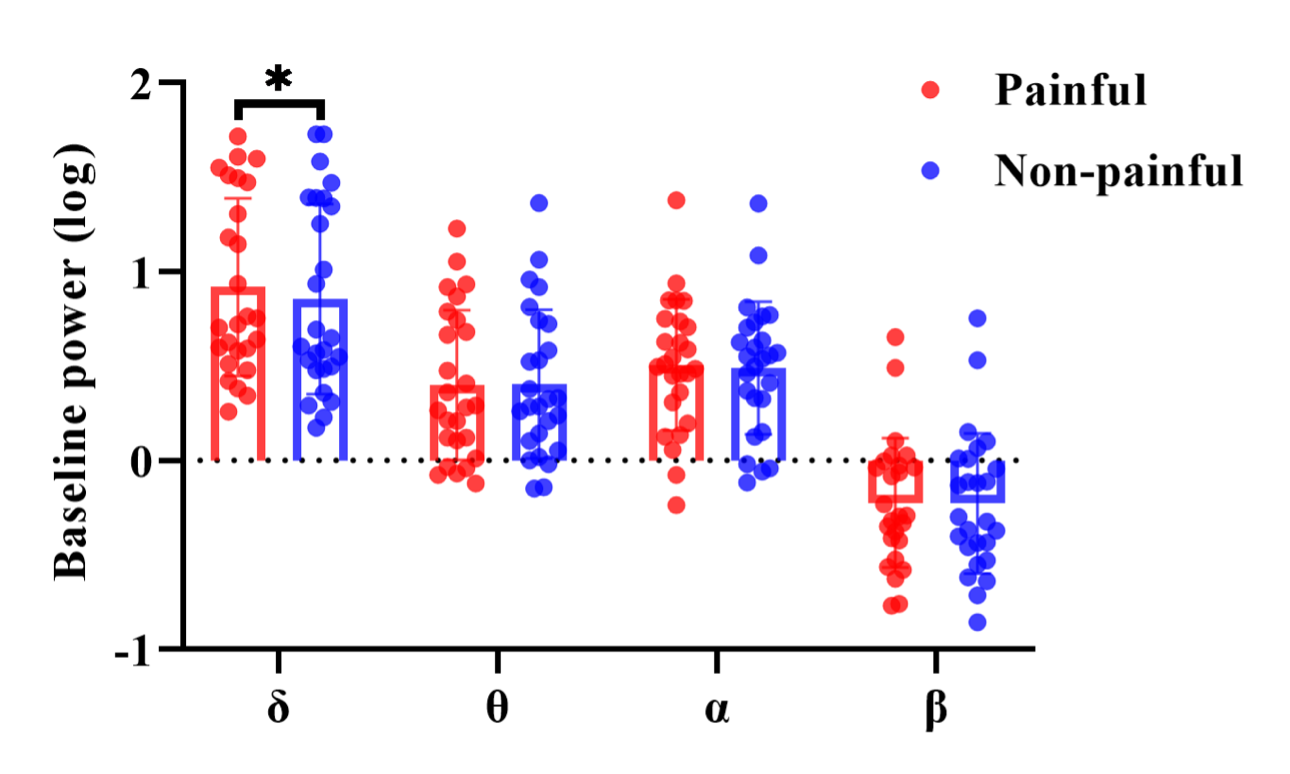


***Figure S1. Baseline power difference between the two types of electrical stimulation.*** *δ(delta)，θ(theta)，α(alpha)，β(beta)；* represents p < 0.05.*
